# Healing Initial of Appendectomy Infection Wounds With Aggressive Washing Method

**DOI:** 10.5812/ircmj.6552

**Published:** 2013-09-05

**Authors:** Iraj Feizi, Iraj Poorfarzan, Bita Shahbazzadegan, Firouz Amani

**Affiliations:** 1 Ardabil University of Medical Sciences, Ardabil, IR Iran

**Keywords:** Wound Infection, Appendectomy, Appendicitis

## Abstract

**Background:**

Appendicitis is one of the most common causes of mortality, despite medical advances it continues to be a major problem.

**Objectives:**

The main goal of this study was healing initial of appendectomy infection wounds with aggressive washing method.

**Patients and Methods:**

This study is a semiexperimental investigation which was performed on 300 patients with perforated appendicitis and infected ulcers who were selected randomly during 2001-2005. Patients were investigated with aggressive washing and primary repair, and necessary data was collected and analyzed.

**Results:**

From all patients, 284 were improved, and 16 cases were complicated, from them 10 patients in the first week, 4 patients in the second week, and 2 patients in the third week had ulcer infection.

**Conclusions:**

The results showed that aggressive washing method is an effective technique in patients with perforated appendicitis and wound infection.

## 1. Background

Appendicitis is one of the most common causes of mortality throughout life, and despite medical advances it continues to be a major problem.

Acute appendicitis is the most common cause of acute abdomen pain in patients admitted to hospitals. By early and true diagnosis, appendectomy is better achieved; ;otherwise, patient may be injured or even death may occur (1). Appendicitis is known a disease for youth and about 40% of cases occur between ages 10 to 29 years. During the life, 12% of men, 25% of women, and about 7% of all people undergo appendectomy for acute appendicitis (2). The most common abdominal surgery in general surgery sections is appendectomy. Due to high prevalence and simple surgical techniques it seems to be less important but could have some problems for patients. Problems that faced Surgeons' with infectious wound Complications in which surgeons are faced with regarding infected wounds include abscess and wound infection which are the most important complications of appendectomy (3).

Abdominal pain associated with nausea and anorexia and in abdominal examination, tenderness and rebound tenderness are the common findings. If the appendicitis is perforated, abdominal pain is extreme and dispersed and the temperature increases to 39 - 40. In this case, patient is unwell and clinical signs are clearly worse (4). Appendicitis has many natures and differential diagnoses. Surgery is the treatment of choice for acute appendicitis and early doing surgery would lead to less complications (5). Actions before and after the operation, have important role in the treatment of acute appendicitis. Lally study showed that prescription of antibiotics before the operation is effective in reducing infectious complications (6). Despite the invention of complex diagnostic equipment, the importance of early surgical intervention should not be ignored (2).

In conventional surgery of perforated appendicitis, after the operation, patient's wound is left open and daily washing is performed, and is closed in the operation room after a delay. This procedure leads to increased complications, duration of hospitalized and patient´s therapeutical costs which are not cost-effective.

## 2. Objectives

So, finding new methods to faster healing of infectious wounds, reducing these complications, and also avoiding high therapeutic costs to patients are of great importance. Therefore the aim of this study was to investigate the aggressive cleaning method for healing infectious wounds instead of commonly used methods for perforated appendicitis.

## 3. Patients and Methods

In this quasi-experimental study, 300 patients admitted to surgery units of Ardabil Fatemi hospital by clinical diagnosis of appendicitis were studied in 2002-2006. In this study, Inclusion criteria were age between 5 to 50 years, no history of diseases such as diabetes, advanced heart disease, obesity, using corticosteroids, and immunosuppressive drugs.

In this study, instead of a two-step and delay healing method, appendectomy infectious wounds were cleaned by aggressive method and repaired in one stage. 

In this method, after facial closing, sub scare washed with 1 - 1.5 liter normal saline by pressure and the initial repair was performed.

Space of operating room, shave, prep, and drip were the same for all patients and monitored by researcher.

Patients were followed up for three weeks after the operation, and occurrence of septic secretion, erythematic, indurations, and high sensitivity was diagnosed as an infection wound by the researcher opinion.

## 4. Results

105(35%) of all patients were female and the rest were male. The mean age of patients was 21 ± 10 and most of them were in the age group of 16-35 years (Table 1). 

**Table 1.** Distribution of Perforated Appendicitis by Age

Age / Group	Number	Percentage
**< 15**	65	21.6
**16 - 35**	158	52.8
**36 - 50**	77	25.6
**Total**	300	100

The rate of perforated appendicitis in rural patients was more than urban patients. 28.5% of all perforated appendicitis patients have used drugs before admission to hospital. 91% of patients had Leukocytosis (WBC > 1000) in laboratory findings, and abdominal pain with tender in right lower quadrant (RLQ) was present in all patients.

Results showed that 284(94.6%) of patients treated with the new method had no sign of infectious wound; while, 10 patient had complications in the first week, 4 patient in the second week, and 2 patient in the third week.

## 5. Discussion

Results showed that washing appendicitis infectious wounds with aggressive wash method decreases the rate of infectious wound significantly compared to the usual method (P = 0.001).

In Fekrat and Kashanian study (2004), sub scare wash with normal saline was known as an effective, cheep, simple and safe method which could decrease the rate of infectious wound which is similar to our study (7). Also, Valent et al. showed that the rate of infectious in wounds washed with normal saline is lower which is similar to our study result (8). Davoudabadi et al. showed that sub scare wash with Ampicillin compared to normal saline had no role in prevention of infectious (9).

Marbery et al. showed that the rate of infections wound after washing with normal saline was decreased, which is similar to our study result (10). Cervantes et al. showed that washing wounds with syringe by pressure in sub scare tissues has significant decreasing role in infectious wounds (11).

Results showed that the primary repair of appendectomy infectious wounds by aggressive washing method could be a better approach versus usual daily wash method due to decreasing hospitalized duration, low therapeutic costs, and complications, and also more healing in patients. 
